# The buffering effect of relationship satisfaction on emotional distress in couples

**DOI:** 10.1186/1471-2458-12-66

**Published:** 2012-01-22

**Authors:** Gun-Mette B Røsand, Kari Slinning, Malin Eberhard-Gran, Espen Røysamb, Kristian Tambs

**Affiliations:** 1 Norwegian Institute of Public Health, Division of Mental Health, PO Box 4404, Nydalen, N-0403 Oslo, Norway; 2 National Network for Infant Mental Health, Centre for Child and Adolescent Mental Health Eastern and Southern Norway (R.BUP Oslo), PO Box 4623, Nydalen, N-0405 Oslo, Norway; 3 Health Services Research Centre, Akershus University Hospital, 1478 Lørenskog, Norway; 4 Department of Psychology, University of Oslo, Oslo, Norway

## Abstract

**Background:**

Marital distress and depression frequently co-occur, and partnership quality is associated with depressive symptoms and mental disorders in both men and women. One aim of this study was to investigate the contribution of a set of risk factors for emotional distress among men and women in couples, with a special focus on satisfaction with partner relationship. The most important aim was to investigate the extent to which high relationship satisfaction in couples acts as a buffer against stressful events.

**Methods:**

Pregnant women and their husbands (n = 62,956 couples) enrolled in the Norwegian Mother and Child Cohort Study completed a questionnaire with questions about emotional distress, relationship satisfaction, and other risk factors. Twelve potential risk factors were included in the analyses, including relationship satisfaction, demographic characteristics, and somatic diseases in men and women. Associations between the predictor variables and emotional distress were estimated by multiple linear regression analysis. Cross-spousal effects, in which data reported by one of the spouses predicted emotional distress in the other, were also investigated. Possible interaction effects between certain risk factors and self-reported and partner's relationship satisfaction were tested and further explored with regression analyses in subsamples stratified by relationship satisfaction scores.

**Results:**

The unique effects of relationship satisfaction were of similar sizes for both men and women: substantial for self-reported (β = -0.23 and β = -0.28, respectively) and weak for partner-reported satisfaction (β = -0.04 and β = -0.02, respectively). Other relatively strong risk factors were somatic disease, first-time motherhood, and unemployment. Self-reported as well as partner-reported relationship satisfaction appeared to strongly buffer the effects of a number of stressors.

**Conclusions:**

Partner relationship dissatisfaction is strongly associated with emotional distress in men and women. Good partner relationship, both as perceived by the individual him(her)self and by the spouse, quite strongly moderates adverse effects of various types of emotional strain.

## Background

For many adults, marriage or cohabitation constitutes their most central and enduring social relationship and has been linked to greater life satisfaction, low rates of depression, and a reduced risk of all-cause mortality. Poor relationship quality may compromise both physical and mental well-being [1,2]. Life transitions, such as pregnancy, represent periods in life in which relationship quality may play an important role.

Understanding the mechanisms of the interpersonal context of psychological distress remains a vital area of scientific research. Interpersonal or interactive models of depression [3,4], family systems models of depression [5,6], and models of emotional contagion [7] all suggest that the social context and the partner relationship play a critical role in the creation, transmission, and maintenance of depressive symptoms [8].

Poor mental health-in particular depression-among men and women is a major health problem that not only affects individuals, but also may have severe negative effects on their families [9-11]. Living with a depressed partner is associated with psychological distress and marital strain [12-14].

Depression is the most comprehensively studied mental disorder in terms of its effects on parents and, consequently, on their children. The research literature has consistently documented that mental health problems in one or both parents are associated with an increased risk of psychological and developmental difficulties in children [15], and both genetic factors and shared environment are demonstrated to play a role [16].

### The association between relationship satisfaction and depressive symptoms

A number of studies using a range of designs and samples have documented a robust association between marital dissatisfaction and depressive symptoms in the general population [17,18]. This association has been documented to be valid for both men and women [19-21].

Although suggested by some studies [22], there is relatively little evidence that women are more affected by marital discord than are men [17]. A meta-analysis of the literature on the association between depression and marital discord identified 26 studies assessing depressive symptoms and marital satisfaction. The results showed across these studies a weighted mean correlation between depressive symptoms and marital satisfaction of -0.42 for women and -0.37 for men [23].

Longitudinal studies can reveal more about causal direction than cross-sectional studies. However, the literature is somewhat contradictory regarding longitudinal relations between relationship satisfaction and depressive symptoms. Marital difficulties are both precursors and sequels to depression [24]. Some studies have shown that marital dissatisfaction predicts subsequent depressive symptoms [25] while the opposite has been found in other studies [26]. In the face of conflicting results, one review of the literature concluded that the associations between depression, relationship satisfaction, and third variables are most likely bidirectional [23].

To date, most research on relationship dissatisfaction and psychological distress has been based on small samples that are not necessarily representative of the population of married or cohabiting individuals [27]. Clearly, results from larger population-based samples are needed.

### Other risk factors associated with symptoms of depression

Depression symptom levels are associated with psychosocial factors like job loss, money problems, and social isolation [28], unfavorable socioeconomic circumstances [29], and poor physical health and low social support [30]. Low self-esteem is another well-known risk factor associated with depression, especially for men [31]. Among women expecting a child, studies show that first-time motherhood may be associated with increased risk for depression, see e.g. [32,33].

Life transitions may represent vulnerable periods for mental health problems [34]. Pregnancy is one common life transition. With the exception of factors associated specifically with pregnancy, like first-time motherhood, the risk factors for depression in pregnant women (see e.g. [35]) seem to be much the same as for the general population [17,18,28-30]. Less is known about prevalence and risk factors for depressive symptoms in men when a new child is expected. One study investigated risk factors for psychological distress in 327 couples from mid-pregnancy to four months after childbirth. Among variables strongly related to male distress were low emotional support from the partner and low dyadic adjustment. For women, low emotional support from friends and low dyadic adjustment were strong predictors. However, this investigation gives no clear evidence of gender differences [36]. Another study based on a sample of 687 women and their partners found that most predictive factors of depression during pregnancy were similar for both genders, but the impact of social support and partner depression appeared to be more important for men than for women [37]. Severe anxiety and depression during pregnancy have been associated with poor pregnancy outcomes, such as preterm delivery, low infant birth weight, and small-for gestational-age infants [38]. A recent longitudinal study showed that antenatal depression related more strongly to negative child outcomes measured in the early school years than did maternal depression at any time postpartum [39]. Emotional distress, particularly depression, is also associated with reduced quality of parenting [40,41].

In conclusion, a number of risk factors have been identified. However, most of the research, particularly when it comes to pregnant couples, is based on small samples with limited power to obtain precise estimates and to investigate interaction effects.

### Cross-spousal effects

Researchers have examined the extent to which the levels of well-being and health of respondents can predict the mental health of their spouses, so-called "cross-spousal effects."

Some studies have focused on the association between depressive symptoms in spouse pairs, and indicated that the depressive symptoms of one spouse influenced those of the other [7]. Other studies have found that one spouse's physical health problems were associated with the other's depressive symptoms [42,43]. To our knowledge, no previous large-scale study has investigated the direct effect of one spouse's relationship satisfaction on the other's emotional distress. Our data set permit the observation of such cross-spousal effects.

### Buffering effects of relationship factors

In addition to the strong main effects of relationship satisfaction on mental health that has previously been demonstrated [17-21], a good relationship may also have an additional protective effect under otherwise stressful conditions. In accordance with the buffering hypothesis [44,45], some factors may protect against severe effects of certain strains. Social support has been shown to be an important protective factor regarding an individual's ability to handle and recover from stressful events [46,47]. Satisfaction with the partner relationship may also be a protective factor against strain. One study examined the relationship between marital quality, onset of depression, and gender following a severely threatening life event and found that a satisfying marriage was related to lower rates of depression for both men and women, although the overall rate for women was higher [48]. A study of the present sample has already demonstrated a strong negative relationship between self-reported relationship satisfaction and emotional distress in women [49]. The results also showed a buffering effect of relationship satisfaction on the effects of some risk factors. Thus, some types of stress will probably be more tolerable for persons who feel content with their partner relationship.

### Aims of the present study

The first aim was to identify own and spousal risk factors for emotional distress in male and female partners. We estimated the contribution of 12 well-known risk factors, observed for both spouses, including the following variables: relationship satisfaction, self-esteem, socio-demographic characteristics, unemployment, somatic disease, social support, and first-time motherhood. Consistent with previous research, our main hypothesis was that relationship satisfaction would be of particular importance --and to a similar extent for men and women--for symptoms of anxiety and depression. Our expectations were strengthened by the fact that the couples were expecting a child. The data structure permitted estimation of cross-spousal effects in addition to the effects of self-reported variables for each individual. We extended this aim by asking to what extent the partner's relationship satisfaction is associated with emotional distress beyond the association between own relationship satisfaction and emotional distress. We also examined the possible cross-spousal effects of factors such as the partner's socio-demographic variables, somatic diseases, and self-esteem.

The second, and most important, aim was to explore to what extent high levels of partner relationship satisfaction, both self-perceived and as reported by the partner, buffer against adverse psychological effects of certain strains. We hypothesized that relationship satisfaction could be a protective factor for both genders. This hypothesis was tested by examining possible interaction effects between relationship satisfaction and certain risk factors on emotional distress in men and women.

## Methods

### Participants

This study is based on the Norwegian Mother and Child Cohort Study (MoBa) conducted by the Norwegian Institute of Public Health [50]. In brief, MoBa is a cohort of more than 100,000 pregnancies recruited from 1999 to 2009 and presents a broad basis to study health development. There were no exclusion criteria, and all maternity units (except two) in Norway with more than 100 births annually were included during certain periods. Mothers undergoing their first routine ultrasound examination, performed at gestation week 17-18, were invited to participate together with their male partners. The women received a postal invitation to participate in the MoBa together with their appointment cards for the ultrasound scan http://www.fhi.no/morogbarn. More than 90% of fathers accompanied their partner to this examination, and the fathers were then asked to take part in the study. A total of 90,190 women (38.7% of the invited women) and 71,648 men (30.5%) participated. There were valid data for both spouses in 66,888 couples. Missing data were imputed (see below), but for some cases too much of the information was missing, preventing reliable imputation, leaving us with 62,956 couples with valid data.

Of the couples responding to the questionnaire, 49.2% were married and the vast majority of the others were co-habiting partners. The women, but not the men, were also followed up at later times [50], so far with seven data collections during a time-span of nine years. Further follow-ups are planned.

The current study is based on Version 4 of the quality-assured data files released for research in 2008. Informed consent was obtained from each participant, both men and women. The study was approved by The Regional Committee for Medical Research Ethics and the Norwegian Data Inspectorate.

When couples completed the first questionnaire, the mean age was 29.7 years (SD = 4.6) for women and 32.2 years (SD = 5.4) for men. There are some differences in descriptive statistics between the MoBa participants and the total population of Norwegian mothers. A recent study found an underrepresentation of the youngest women (< 25 years old), those living alone, mothers with more than two previous births, and those with previous stillbirths. Despite this, no statistically relevant differences in association parameters between participants and the total population were found regarding a number of exposure-outcome associations [51]. The sample has been described in more detail elsewhere [50,51].

### Measures

The women and men completed different questionnaires, but most of the items on mental health were common to the two questionnaires.

#### Emotional distress

Male and female emotional distress was measured using a short version of the Hopkins Symptom Checklist (SCL-25) [52]. The SCL is a self-administered instrument designed to measure symptoms of anxiety and depression [53]. The five-item version (SCL-5) correlates 0.92 with the original version [54]. The sum of the five anxiety and depression items was treated as a global measure of mental health, hereafter termed emotional distress. The SCL-5 [54] consists of the following items: Have you been bothered by any of the following during the last two weeks: 1) Feeling fearful; 2) Nervousness or shakiness inside; 3) Feeling hopeless about the future; 4) Feeling blue; or 5) Worrying too much about things?" The response categories are 1 = not at all, 2 = a little, 3 = quite a bit, and 4 = extremely.

The Cronbach alpha reliability for the SCL-5 was 0.81 for both women and men. The distribution of the SCL-5 scores was highly skewed with a tail to the right. Therefore, the SCL-5 scores were logarithm-transformed to approximate a normal distribution. Skewness was reduced to 1.24 and kurtosis to 1.07. The dependent variable was standardized before inclusion in the analyses.

#### Relationship satisfaction

The 10-item Relationship Satisfaction (RS10) scale [55] constructed for MoBa and based on typical items used in previously developed scales [56,57] was used to measure perceived partner relationship satisfaction. The RS10 scale shows good psychometric properties, with a Cronbach alpha = 0.91, correlates 0.92 with the Quality of Marriage Index [58], and in general shows high structural and predictive validity [55]. The scale contains 10 items, such as "I am satisfied with the relationship to my partner" and "My husband/partner and I have a close relationship." The response categories range from 1 (strongly disagree) to 6 (strongly agree). A total of 29,265 men completed a questionnaire version with a short version of the RS scale, which consisted of five out of ten items (RS5). The five-item version has previously been shown to correlate at 0.97 with the full version [55]. We generated imputed values for men with values on five or more items, regardless of how many RS items they had been asked to complete. An indicator of overall relationship satisfaction based on 10 items was computed as an average score across items. The Cronbach alpha reliability was 0.90 for women and 0.91 for men for the RS scale.

#### Self-esteem

A 4-item short version of the Rosenberg Self-Esteem Scale (RSE) [59] tapped global self-esteem. A sum of the four items correlates at 0.95 with the score from the original instrument, and the Cronbach alpha is 0.80. This instrument is described elsewhere [60].

#### Social support

One question about social support was included in the analyses. This item measured the number of supportive persons in addition to the partner: "Do you have anyone other than your partner whom you can ask for advice in a difficult situation?" There were three response categories: "no", "yes, 1-2 people", and "yes, more than 2 people." Because the questionnaire completed by the men was modified during the study, only 29,265 men were asked this question. Therefore, the question about mens' social support was used only in supplementary analyses of interaction effects. The social support indicators were standardized before inclusion in the analyses.

#### Somatic diseases

Information on women's somatic diseases was obtained using a checklist of 53 symptoms/illnesses covering seven different groups of diseases: asthma/allergy/eczema, diabetes, cardiovascular disease/high blood pressure/hyperthyroidism/hypothyroidism, gastrointestinal disease, muscular/skeletal/articular disease, gynecological/urinary/kidney disease, and "other disease". The respondents reported whether or not they had experienced problems in these areas before or during pregnancy.

Seven summative indices were created using the summed scores for each of the seven groups of diseases. The seven indices were entered into a regression analysis with women's emotional distress as a dependent variable. Then a general indicator based on the seven disease-group scores was generated to estimate the overall effect of somatic disease. The scores for each separate disease group were weighted by their respective regression coefficients estimated in the initial analyses and then summed. This procedure maximizes the predictive power of the global somatic indicator for mental health. When the original seven somatic items are replaced by this index in the principal multivariate analysis of predictors of mental health, the variance explained by somatic diseases is essentially unchanged. The purpose of collapsing these predictors is to obtain one single estimate of the total effect of all somatic diseases. The somatic disease indicator was standardized before inclusion in the analyses.

Information on men's somatic diseases was obtained using a checklist of 19 symptoms/illnesses covering eight different groups of diseases: asthma/allergy, skin disease/eczema, diabetes, cardiovascular disease/high blood pressure, gastrointestinal disease, muscular/skeletal/articular disease, urinary/kidney disease, and "other disease." The participants reported whether they had previously experienced problems in these areas or do so currently. We used the same procedure for men as described for women to obtain one single estimate of the total effect of somatic diseases. The male somatic disease indicator was standardized before inclusion in the analyses.

#### First time motherhood

One dichotomous variable measuring first-time motherhood (no = 0, yes = 1) was included in the analyses.

#### Sociodemographic variables

Sociodemographic variables included total family income (measured using the combined income for the woman and the man scored from 0 (= no income) to 12 (≥ NOK 1 M ≈ USD 168,000), educational level (six categories from public school to > 4 years at university/college), and unemployment (disability retirement, or out of work). The item measuring unemployment was coded as a dichotomous variable (no = 0, yes = 1).

### Treatment of missing values

#### Rates of missing values

The frequency of missing values across variables used in the analyses varied between 0.86% and 5.2%, with an average of 2.6%. Because of different versions of the male questionnaire, a total of only 42,383 men were asked to fill in a ten-item RS scale; the others completed a five-item version. The correlation between the two versions, observed in the subsample with ten items, was 0.96. There were incomplete data in 3.7% of the male ten-item RS responses and 2.2% of the male five-item responses. Imputed values were generated only for responses with valid data on five or more items, regardless of the ten- or five-item version.

#### Replacement of missing values

The analyses included a large number of predictor variables, which together produced a large number of cases with at least one missing value. Therefore a relatively liberal treatment of missing values was necessary to prevent a substantial and possibly non-random loss of data. We used SPSS MVA, Expectation Maximization [61] to impute values for missing scores on the continuously distributed scales SCL-5, RS, and RSE. The imputations were conducted separately for each scale, using the remaining scale items to predict values replacing missing values. Imputed values were generated if respondents already had valid data for at least half of the items on the scale.

The correlations between income and education and between those and other variables were too low to gain much information by imputation in a predictive manner. Nonetheless, non-response on income was associated with low education and vice versa, indicating that missing values for these variables are highly non-random and that most of the lost observations are from the lower parts of the distributions. Here, neither EM imputation nor regular mean substitution appeared to be suitable. Instead mean values on valid demographic data for non-responders compared to responders for a particular variable were used to choose a suitable constant for replacing missing values for female and male income and education.

We used female age and female and male income and education, which are all inter-correlated, to calculate suitable constants by which missing values on education and income were replaced. The items on male and female education in addition to six categories (scored 0-5) had one category for 'other education'. Values on income and partner's education for subjects checking "other education" indicated that the best values to replace "other education" were 3.5 (3 = high school) for women and 4 (university/college, < 4 years) for men. Low mean income values for men and women not responding to the education question indicated that the best replacement for missing education data was the lowest score. Missing values on men's income (mean = 3.93, SD = 1.36) and women's income (mean = 3.02, SD = 1.33) were replaced by the constants 3.5 and 2.5, respectively, before summed to give a measure of total family income.

Among the 90,190 women and 71,648 men initially recruited to participate in the study, there were 66,888 complete couples. However, for 6,542 (7.3%) of the women and 6,586 (9.2%) men it was not possible to impute one or more key study variables resulting in a final, usable sample of 83,648 women and 65,062 men who comprised 62,956 usable couples.

### Statistical analyses

The 12 selected predictors of maternal and paternal emotional distress measured in the 17th gestational week were examined using multiple regression analyses. The data set was analyzed twice. First with the women's emotional distress as the dependent variable and the women's and their partners' self-reported data as independent variables. Then, these analyses were rerun with the men's emotional distress as the dependent variable. Personal self-esteem was not entered in the main analyses because of the high content overlap with the dependent variable.

We tested for possible interaction effects between RS scores and other predictors by including the product of the interacting variables in the regression analysis. Interaction effects were tested in separate regression analyses together with all the predictors, one interaction term at a time. Examinations of interaction effects on maternal and paternal emotional distress were conducted for both self-reported and partner's relationship satisfaction with each of the 10 other independent variables. Significant interaction effects were further examined by stratifying the sample on the relationship satisfaction variable and repeating the regression analyses in each stratum. We stratified the RS scores into three groups: low RS (score 1-3.99, including 3.4% of the women and 2.6% of the men), moderate RS (score 4-4.99, including 15.6% of the women and 17.1% of the men), and high RS (score 5-6, including 80.9% of the women and 80.3% of the men). Self-esteem (both personal and partner's) was included in the interaction analyses, since the content overlap with the dependent variable does not seriously affect the results from the interaction analyses. For all the analyses, *p *< 0.001 was used as significance level, due to the large number of tests and the large sample size.

## Results

### Descriptive statistics

The mean score on the SCL-5 was 1.25 (SD = 0.38) for women and 1.13 (SD = 0.29) for men. The correlation between the partners' SCL-5 scores was 0.19. The mean score on the RS scale was 5.35 (SD = 0.56) for men and 5.36 (SD = 0.60) for women (t = 6.73, *p *< 0.001). The correlation between the partners' RS scores was 0.55. The correlations between all the predictor variables are shown in Table 1.

**Table 1:** Pearson correlations between all the predictors in the analyses

Variable	Mean	SD	**1**.	**2**.	**3**.	**4**.	**5**.	**6**.	**7**.	**8**.	**9**.	**10**.	**11**.	**12**.
**1. RS women**	5.36	0.60												

**2. Self-esteem women**	2.31	0.49	0.35											

**3. Soc.support women**	2.50	0.56	0.16	0.19										

**4. Unemployment women**	0.04	0.20	-0.04	-0.09	-0.05									

**5. Education women**	3.51	1.36	0.05	0.15	0.14	-0.11								

**6. Somatic disease women**	0.11	0.11	-0.06	-0.09	0.01	0.08	-0.01							

**7. Family income**	6.94	2.18	0.05	0.16	0.09	-0.13	0.39	-0.02						

**8. First-time motherhood**	0.49	0.50	0.18	0.02	0.12	-0.02	0.01	0.07	-0.06					

**9. RS men**	5.35	0.56	0.55	0.20	0.11	-0.03	0.04	-0.03	0.03	0.16				

**10. Self-esteem men**	2.48	0.45	0.19	0.18	0.07	-0.03	0.08	-0.03	0.13	0.01^NS^	0.32			

**11. Unemployment men**	0.03	0.18	-0.05	-0.05	-0.04	0.08	-0.09	0.01	-0.18	0.01	-0.04	-0.10		

**12. Education men**	3.23	1.46	0.09	0.11	0.11	-0.07	0.39	-0.02	0.34	0.00^NS^	0.03	0.12	-0.10	

**13. Somatic disease men**	0.15	0.20	-0.06	-0.05	-0.04	0.08	-0.06	0.07	-0.05	-0.03	-0.07	-0.10	0.10	-0.09

All correlations except those marked NS are significant, *p *< 0.001

### Effects of various risk factors on the level of emotional distress in female and male spouses

All 12 factors were significantly associated with women's emotional distress (SCL-5 score) after mutually controlling for all variables. For men, 9 out of 12 factors had a significant effect. Table 2 shows the factors that had a significant unique effect on female emotional distress. The corresponding results for males are shown in Table 3. SCL-5 was standardized; therefore the non-standardized regression coefficient (b) shows the expected difference in standard deviations in SCL-5 per predictor scale unit (Cohen's d). In most cases, the independent variables in the analyses were also standardized, implying that the b estimates are identical to the β estimates. For dichotomous variables (not standardized) the β estimates are reported in the table subtext.

**Table 2:** Effect of various risk factors on the level of emotional distress (scl-5 score) in 62,956 women

Risk/protection factor	Range (before z-transformation)	% exposed	Crude b	Adjusted b^f^	95% CI
Relationship satisfaction^a^	1-6		-0.31	-0.28	-0.29,-0.27

Partner's relationship satisfaction^a^	1-6		-0.19	-0.02	-0.03,-0.01

Partner's self-esteem^a^	0-3		-0.13	-0.04	-0.05,-0.04

Social support^a^	1-3		-0.12	-0.07	-0.07,-0.06

Family income^a^	0-12		-0.18	-0.09	-0.09,-0.08

Unemployment^b^	0, 1	4.3	0.51	0.27	0.24, 0.31

Partner's unemployment^c^	0, 1	3.4	0.41	0.14	0.10, 0.18

Education^a^	0-5	22.3^e^	-0.14	-0.06	-0.07,-0.05

Partner's education^a^	0-5	22.6^e^	-0.08	0.02	0.02, 0.03

Somatic disease^a^			0.17	0.14	0.13, 0.15

Partner's somatic disease^a^			0.06	0.02	0.01, 0.02

First time motherhood^d^	0, 1	48.8	0.07	0.16	0.14, 0.17

All effects are significant, *p *< 0.001.

^a: ^Z-transformed, SD = 1. For all these risk factors, both the dependent and the independent variables are standardized. Thus, the reported adjusted b's equal Beta.

^b: ^SD = 0.20, β = 0.06, ^c:^SD = 0.18, β = 0.03, ^d:^SD = 0.50, β = 0.08

^e: ^frequency of top score (men's and women's education: more than 4 years university education).

^f: ^All predictors adjusted for all other predictors

**Table 3:** Effect of various risk factors on the level of emotional distress (scl-5 score) in 62,956 men

Risk/protection factor	Range (before z-transformation)	% exposed	Crude b	Adjusted b^f^	95% CI
Relationship satisfaction^a^	1-6		-0.27	-0.23	-0.24,-0.22

Partner's relationship satisfaction^a^	1-6		-0.20	-0.04	-0.05,-0.03

Partner's self-esteem^a^	0-3		-0.11	-0.02	-0.03-0.01

Family income^a^	0-12		-0.12	-0.08	-0.09,-0.07

Unemployment^b^	0, 1	3.4	0.75	0.50	0.46, 0.54

Education^a^	0-5	22.6^d^	-0.04	0.03	0.02, 0.04

Somatic disease^a^			0.20	0.17	0.16, 0.17

Partner's somatic disease^a^			0.05	0.02	0.02, 0.03

First time motherhood^c^	0, 1	48.8	0.03	0.11	0.10, 0.12

All tabulated effects are significant at the *p *< 0.001 level.

The following predictors had no significant effect on scl-5 (*p *> 0.001): Partner's social support, partner's unemployment, and partner's education.

^a: ^Z-transformed, SD = 1. For all these risk factors, both the dependent and the independent variables are standardized. Thus, the reported adjusted b's equal Beta.

^b: ^SD = 0.18, β = 0.09, ^c:^SD = 0.50, β = 0.06.

^d: ^frequency of extreme high score (men's and women's' education: more than 4 years university education).

^f: ^All predictors adjusted for all other predictors

Self-reported relationship satisfaction had a substantial effect on women's emotional distress. First-time motherhood and somatic disease also showed clear effects. There was a substantial effect of unemployment; 4.3% of the women reported that they were unemployed.

As seen in Table 3, relationship satisfaction was also the most important factor for men's emotional distress. Somatic diseases and partner's first-time motherhood (and, in most cases, personal first-time fatherhood) were also substantial factors for men's emotional distress. Unemployment was a particularly important condition among the relatively few (3.4%) men affected.

### Cross-spousal effects

The analyses revealed significant cross-spousal effects on emotional distress in both women and men. For women, partner's self-esteem and partner's unemployment yielded the clearest cross-spousal effects. For men, the spouse's relationship satisfaction and somatic diseases affected emotional distress most.

### Relationship satisfaction as a buffer against emotional distress

Table 4 shows significant interaction effects on women's emotional distress between own relationship satisfaction and the following ten predictors: self-esteem, first-time motherhood, education, somatic disease, social support, family income, partner's emotional distress, partner's relationship satisfaction, partner's unemployment, and partner's education. In general, the results indicated that a perception of high relationship satisfaction protects against the possible negative effects of the risk factors or the absence of the protective factors. No significant interaction effects were found between relationship satisfaction and the following variables/predictors: unemployment, partner's self-esteem, partner's social support, and partner's somatic disease.

**Table 4:** Relationship satisfaction (RS) as a buffer for women.

Relationship satisfaction (own)	Self-esteem (SD-scored)	First time motherhood (0,1)	Education (SD-scored)	Somatic disease (SD-scored)	Social support (SD-scored)	Family income (SD-scored)	Partner's distress (SD-scored)	Partner's RS (SD-scored)	Partner unemployed (0,1)	Partner's education (SD-scored)
Low	-0.40 (-0.44,-0.35)	0.37 (0.26, 0.48)	-0.11 (-0.17,-0.06)	0.13 (0.09, 0.17)	-0.15 (-0.20,-0.11)	-0.10 (-0.16,-0.05)	0.13 (0.09, 0.17)	-0.07 (-0.11,-0.03)	0.19 (-0.02, 0.39)	-0.02 (-0.07, 0.03)

Moderate	-0.37 (-0.39,-0.35)	0.26 (0.21, 0.30)	-0.10 (-0.12,-0.08)	0.16 (0.14, 0.18)	-0.07 (-0.09,-0.05)	-0.12 (-0.15,-0.10)	0.12 (0.10, 0.14)	0.00 (-0.02, 0.02)	0.16 (0.06, 0.26)	0.05 (0.03, 0.07)

High	-0.29 (-0.30,-0.28)	0.12 (0.11, 0.14)	-0.05 (-0.06,-0.04)	0.14 (0.13, 0.15)	-0.06 (-0.07,-0.06)	-0.08 (-0.09,-0.07)	0.12 (0.11, 0.13)	-0.02 (-0.03,-0.01)	0.12 (0.07, 0.16)	0.02 (0.01, 0.03)

Significant interaction effects (*p *< 0.001) between relationship satisfaction and ten predictors on women's emotional distress. Main effects (b (95% CI)) for various strata with low, moderate, and high relationship satisfaction

No significant interaction effect (*p *> 0.001) was found for 'Unemployment X Relationship satisfaction', Partner's self-esteem X Relationship satisfaction', Partner's social support X Relationship satisfaction' and Partner's somatic disease X Relationship satisfaction'

As shown in Table 5, significant interaction effects on men's emotional distress were found between own relationship satisfaction and the following nine predictors: self-esteem, first time motherhood, unemployment, somatic disease, family income, partner's emotional distress, partner's self-esteem, partner's relationship satisfaction, and partner's social support. In general, the results indicated that high relationship satisfaction protects against the possible negative effects of these nine variables in men. No interaction effect was found between subjective RS and the following predictors: social support, education, partner's unemployment, partner's somatic disease, and partner's education.

**Table 5:** Relationship satisfaction (RS) as a buffer for men.

Relationship satisfaction (own)	Self-esteem (SD-scored)	First time motherhood (0,1)	Unemployment (0,1)	Somatic disease (SD-scored)	Family income (SD-scored)	Partner's distress (SD-scored)	Partner's self-esteem (SD-scored)	Partner's RS (SD-scored)	Partner's social support
Low	-0.53 (-0.58,-0.47)	0.29 (0.13, 0.45)	0.59 (0.28, 0.90)	0.20 (0.14, 0.25)	-0.21 (-0.29,-0.13)	0.15 (0.08, 0.22)	-0.01 (-0.08, 0.06)	-0.11 (-0.16, -0.05)	-0.06 (-0.13, 0.01)

Moderate	-0.36 (-0.38,-0.34)	0.16 (0.12, 0.21)	0.53 (0.42, 0.64)	0.22 (0.20, 0.24)	-0.11 (-0.14,-0.09)	0.16 (0.13, 0.18)	-0.04 (-0.06,-0.01)	-0.01 (-0.03, 0.01)	0.00 (-0.03, 0.02)

High	-0.25 (-0.25,-0.24)	0.09 (0.07, 0.10)	0.48 (0.44, 0.52)	0.15 (0.14, 0.16)	-0.07 (-0.08,-0.06)	0.13 (0.12, 0.14)	-0.02 (-0.03,-0.01)	-0.03 (-0.05,-0.02)	0.00 (-0.01, 0.00)

Significant interaction effects (*p *< 0.001) between relationship satisfaction and nine predictors on men's emotional distress. Main effects (b (95% CI)) for various strata with low, moderate, and high relationship satisfaction

No significant interaction effect (*p *> 0.001) was found for 'Social Support X Relationship satisfaction', 'Education X Relationship satisfaction', 'Partner's unemployment X Relationship satisfaction', Partner's somatic disease X Relationship satisfaction', 'Partner's education X Relationship satisfaction'

### The partner's relationship satisfaction as a buffer against emotional distress

Table 6 shows that significant interaction effects on women's emotional distress were found between the partner's relationship satisfaction and the following seven predictors: self-esteem, first time motherhood, education, social support, subjective relationship satisfaction, family income, and unemployment.

**Table 6:** Partner's relationship satisfaction as a buffer for women.

Relationship satisfaction (partner's)	Self-esteem (SD-scored)	First time motherhood (0,1)	Education (SD-scored)	Social support (SD-scored)	Relationship satisfaction (SD-scored)	Family income (SD-scored)	Unemployment (0,1)
Low	-0.38 (-0.43,-0.33)	0.35 (0.23, 0.47)	-0.14 (-0.20,-0.08)	-0.15 (-0.20,-0.10)	-0.35 (-0.38,-0.31)	-0.03 (-0.09, 0.03)	0.29 (0.07, 0.52)

Moderate	-0.35 (-0.37,-0.34)	0.24 (0.21, 0.28)	-0.08 (-0.10,-0.06)	-0.08 (-0.10,-0.06)	-0.31 (-0.33,-0.29)	-0.11 (-0.13,-0.09)	0.34 (0.25, 0.42)

High	-0.29 (-0.30,-0.28)	0.13 (0.11, 0.14)	-0.05 (-0.06,-0.05)	-0.06 (-0.07,-0.05)	-0.25 (-0.26,-0.24)	-0.09 (-0.10,-0.08)	0.25 (0.21, 0.29)

Significant interaction effects (*p *< 0.001) between the partner's relationship satisfaction and seven predictors on women's emotional distress. Main effects (b (95% CI)) for various strata with low, moderate, and high relationship satisfaction

No significant interaction effect (*p *> 0.001) was found for 'Somatic disease X Partner's relationship satisfaction' 'Partner's self-esteem X Partner's relationship satisfaction', 'Partner's unemployment X Partner's relationship satisfaction', Partner's somatic disease X Partner's relationship satisfaction', 'Partner's education X Partner's relationship satisfaction', 'Partner's social support X Partner's relationship satisfaction', 'Partner's distress X Partner's relationship satisfaction'

Significant interaction effects on men's emotional distress were found between the partner's relationship satisfaction and the following six predictors: self-esteem, first time motherhood, relationship satisfaction, somatic disease, family income, and partner's emotional distress (Table 7). All except one of the significant effects were found between self-reported--not partner-reported--variables and the partners' perceived relationship quality.

**Table 7:** Partner's relationship satisfaction as a buffer for men.

Relationship satisfaction (partner's)	Self-esteem (SD-scored)	First time motherhood (0,1)	Relationship satisfaction (SD-scored)	Somatic disease (SD-scored)	Family income (SD-scored)	Partner's distress (SD-scored)
Low	-0.40 (-0.45,-0.36)	0.23 (0.10, 0.35)	-0.38 (-0.42,-0.34)	0.20 (0.16, 0.25)	-0.17 (-0.23,-0.11)	0.17 (0.12, 0.22)

Moderate	-0.36 (-0.39,-0.34)	0.18 (0.13, 0.22)	-0.26 (-0.28,-0.24)	0.20 (0.18, 0.22)	-0.13 (-0.16,-0.11)	0.14 (0.12, 0.16)

High	-0.25 (-0.26,-0.24)	0.09 (0.07, 0.10)	-0.20 (-0.21,-0.19)	0.16 (0.15, 0.17)	-0.07 (-0.08,-0.06)	0.13 (0.12, 0.14)

Significant interaction effects (*p *< 0.001) between the partner's relationship satisfaction and six predictors on men's emotional distress. Main effects (b(95% CI)) for various strata with low, moderate, and high relationship satisfaction

No significant interaction effect (*p *> 0.001) was found for 'Social support X Partner's relationship satisfaction', 'Unemployment X Partner's relationship satisfaction', 'Education X Partner's relationship satisfaction', 'Partner's self-esteem X Partner's relationship satisfaction', 'Partner's social support X Partner's relationship satisfaction','Partner's unemployment X Partner's relationship satisfaction', 'Partner's somatic disease X Partner's relationship satisfaction', and 'Partner's education X Partner's relationship satisfaction'

The general trend was for the strongest effects of the risk or protective factors to be evident when the partner was most dissatisfied with the partner relationship. Two exceptions were the effects of unemployment and of family income in women, which tended to be strongest when the partner was moderately satisfied with the relationship.

**Figure 1:**
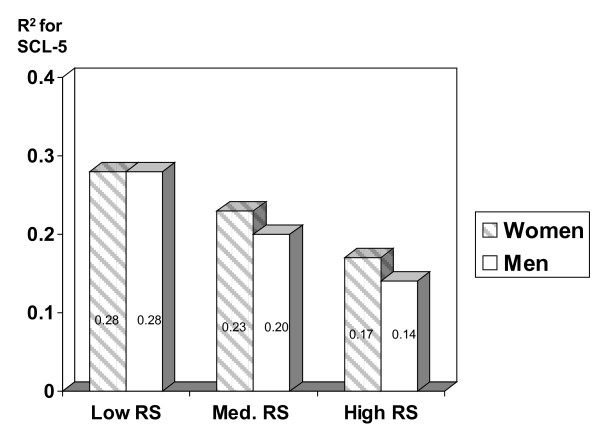
**Total effect of all risk factors (R^2^) on emotional distress (SCL-5) in three levels of *self-reported *RS**.

**Figure 2:**
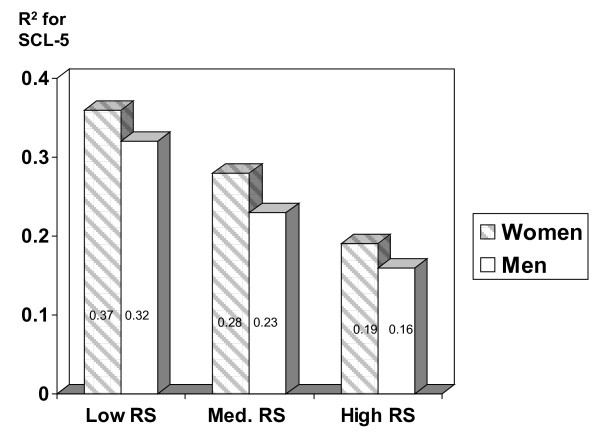
**Total effect of all risk factors (R^2^) on emotional distress (SCL-5) in three levels of *partner-reported *RS**.

Figure 1 illustrates the total effect (R^2^) of all risk factors in strata with low, medium, and high self-reported relationship satisfaction. Figure 2 shows the corresponding effect for strata with different levels of partner-reported relationship satisfaction. The differences between strata in total effects were even stronger for partner's relationship satisfaction as a buffer (Figure 2) than for self-reported RS as a buffer (Figure 1).

A part of the male sample completed a shorter version of the RS scale with only 5 items. We wanted to test whether this instrument abbreviation could have affected the results. We tested for an interaction effect between the RS score and a variable indicating whether the men had completed the RS10 or the RS5 version. There was no interaction effect, showing that the replacement of the full with the short RS scale has not changed the results.

## Discussion

To our knowledge, no other large-scale studies have included a large number of risk factors for emotional distress in couples. The current population based sample with 62,956 spouses gives a statistical power which permits the detection of even trivial main effects and even moderately high interaction effects.

The first aim of our study was is to investigate the relative contribution of a selection of well-known risk factors for emotional distress in 62,956 Norwegian couples. As expected, the 12 predictors included in the analyses all had significant unique effects on women's emotional distress. For men, 9 out of 12 factors had significant unique effects. Relationship dissatisfaction was the strongest predictor for both men and women, and this predictor explained 7.8% of the total variance (in terms of squared adjusted β's) in women and 5.3% of the total variance in men. This finding is in accordance with our hypothesis that relationship satisfaction is of special importance for both genders. Somatic disease, unemployment, and first-time motherhood were also among the strongest predictors for both sexes. Cross-spousal effects were generally weak. The spouse's self-esteem yielded the strongest effect on women's emotional distress, while the spouse's relationship satisfaction was most important for men.

The most important aim was to explore to what extent high levels of relationship satisfaction, perceived both by the individual and the partner, could buffer against adverse psychological effects of certain strains. The results indicated that a subjective feeling of having a good relationship with a partner may protect against the effects of most types of stressors. If the spouse experiences high relationship satisfaction, this may also act as a buffer against certain strains, and whereas the main effect of spousal relationship satisfaction was weak, the buffering effect was remarkably strong. These results are in accordance with our second hypothesis.

### Level of relationship satisfaction in couples

The alpha reliability on the RS scale was 0.90 for women and 0.91 for men. The "true" partner correlation, corrected for imperfect reliability, is 0.55/(0.91^0.5 ^*0.90 ^0.5^) = 0.60, reflecting strong agreement in perceived partnership quality. The majority of the participants in this study were satisfied with their relationship. Most couples plan to have children when their relationship is good and life circumstances such as work and housing feel safe and stable [62]. Under these circumstances, pregnancy is a happy experience associated with positive expectations for most couples. The literature shows a decline in marital satisfaction after pregnancy [63,64], which is what to expect of the time when expecting a child is among the happiest periods in many couples' lives. Also a possible decline could be caused by stress associated with giving birth or with the transition to parenthood.

The low-satisfaction group consisted of slightly more women than men (3.4% versus 2.6%) but the mean values were approximately the same (5.36, SD = 0.60 and 5.35, SD = 0.56). Several studies have found at least some differences in perceived marital quality, typically with women reporting lower levels of self-reported marital quality than men [65,66]. Other studies have found an overall absence of gender differences in marital quality [67,68].

### The effect of relationship satisfaction on emotional distress

As expected, emotional distress was more strongly influenced by personal variables than partner variables for both men and women. Perceived relationship dissatisfaction was the strongest predictor for emotional distress in both genders. This finding has already been demonstrated for women in the present sample [49] and is consistent with previous research on couples [19,21,27].

The results from one meta-analysis of the literature on the association between depression and marital satisfaction showed a weighted mean correlation across studies of -0.42 for women and -0.37 for men [23]. This analysis reported on data from 26 studies involving about 3,700 women and 2,700 men. The correlations from this meta-analysis are about 0.1 higher compared to those in the present study (unadjusted b = -0.31 for women and -0.27 for men). This moderate discrepancy with previous results could partly reflect random fluctuations in rather small sample sizes, perhaps combined with a publication bias in which low or non-significant effects were not accepted for publication.

Some studies have demonstrated that the mental health of wives is more sensitive to relationship factors than the mental health of their husbands [69,70], while others have shown that relationship quality seems to affect the level of psychological distress in similar ways for men and women [71]. The present study showed that partner relationship satisfaction was the most important predictor of emotional distress in both men and women, and the effect sizes were similar across sexes.

### Other risk factors

Somatic disease was among the stronger predictors for emotional distress in both genders. A strong relationship between physical and mental health problems has been demonstrated repeatedly. Individuals with somatic chronic disease frequently suffer from anxiety and depression [72-74].

A minor subgroup of the sample (4.3% of the women, 3.4% of the men) was unemployed, and unemployment proved to be a very strong predictor of emotional distress, more so for men than women. Perhaps as expected during pregnancy, employment was less important for women than for men. A negative link between unemployment and psychological health is well documented [75-77] and previous evidence has also shown stronger effects for men than for women [78]. Our findings are also in line with the results of an earlier study that suggested that unemployment has a greater effect on men's health because of their role as "primary providers for the family," whereas women are protected by their nurturing roles [79]. First-time motherhood was also among the clearest predictors for emotional distress in both genders. Some previous studies have shown that first-time motherhood is a risk factor for depression in pregnant women [32]. Such an effect has not earlier, to our knowledge, been examined for fathers.

Overall--except for unemployment--the results leave an impression that risk factors for emotional distress are mainly the same for men and women.

### Cross-spousal effects

Relationship dissatisfaction, low self-esteem, unemployment, high education, and somatic diseases in the male spouse yielded significant effects on women's emotional distress. Most women feel more vulnerable during pregnancy and may become more dependent upon their partner for emotional and practical support. Pregnancy may be a time when a woman needs more than ever to feel secure about her partner's feelings about her and their relationship and about his emotional and financial ability to take care of her and the new child. Three of the five spousal variables with significant effects on women also predicted emotional distress in men. All the cross-spousal main effects were relatively weak, however.

### Interaction effects

The results also show that experiencing a good partner relationship acts as a protective factor against some significant stressors. Relationship satisfaction had a quite strong buffering effect against low self-esteem and first time motherhood in both men and women and against unemployment and low family income in men.

Although the main cross-spousal effects were generally weak, some of the cross-spousal buffering effects were clearly stronger than trivial. For instance, the partner's relationship satisfaction showed adjusted standardized regression coefficients varying from 0.02 to 0.04, but buffered the effect of own (low) relationship satisfaction from -0.35 to -0.25 in women and from -0.38 to -0.20 in men. Thus, if the partner is satisfied with the relationship, this satisfaction may help both men and women cope better with some strains. In general, the buffering effects sizes did not differ much between women and men.

### Strengths and limitations

High statistical power due to the large number of participants and precise estimates are among the most important strengths of this study. Small effects and even negative results are still highly informative because of the narrow confidence intervals. Some of the effect sizes are relatively small even if they are significant. This study reports on many risk factors, both in male and female spouses, in a critical period of life and the relationship. The large sample also makes it possible to detect interaction effects.

Nonetheless, our findings must be interpreted carefully because of some limitations. First, in cross-sectional studies, data are not informative regarding causal directions. The associations between relationship satisfaction and emotional distress are probably bidirectional. Therefore some of the estimates may be inflated by a reversed effect from poor mental health to relationship satisfaction and to other variables modelled as causal factors. This possible bias effect is less of a problem for the estimated buffering effects than for the main effects, however. For instance, if poor mental health affects social support, and thus inflates our estimates of the effects of social support on mental health, it probably does so essentially independently of relationship satisfaction. Accordingly, even if somewhat inflated, the effect estimates for social support in different strata of relationship satisfaction can still be compared.

Second, as in all studies based on questionnaires, there may be response biases that cause spurious correlations between self-reported predictor- and outcome variables. Third, the validity and reliability of the outcome measure and some of the predictor variables might be less than optimal. For instance social support is measured with a single item. Whereas the first and second limitation could have led to inflated estimates, the third could have deflated them. Fourth, the response rates were low. However this is not uncommon in large epidemiologic studies and does not necessarily imply an unrepresentative sample [80]. Also, while preventing reliable estimation of the occurrence of mental health problems, a moderate sample selection is not expected to dramatically affect results from analytic epidemiology [80]. Nevertheless, our results may have been somewhat biased due to sample selection. The most likely type of bias would be a moderate attenuation of the effect estimates due to restricted variance both for relationship satisfaction and for psychological distress.

Our sample consists of couples in a certain phase of life. Consequently, we do not know the extent to which the results can be generalized to other samples of couples. These pregnant couples may be in a vulnerable phase of life, in which the protective effect of a good relationship may be extra strong. Comparisons with future results from large scale normal population studies will show. Nonetheless, we still believe that these findings, showing the importance of a satisfying relationship, have implications for the population in general.

## Conclusion

These findings demonstrate the importance of the quality of the partner relationship in addition to first-time motherhood, somatic disease, and unemployment as predictors of emotional distress in men and women. By revealing cross-spousal effects on emotional distress, this study contributes to the understanding of how partners influence each other's emotional distress. In addition, relationship satisfaction appeared to strongly buffer the effects of certain strains for both men and women. Thus, when an adult seeks help for depressive symptoms, the partner relationship is an important consideration in treatment.

Based on earlier research on the association between relationship satisfaction and emotional distress, we suppose that these findings are valid for most couples, regardless of pregnancy. Nevertheless, it is, for several reasons, particularly important to identify and support women and men suffering from emotional distress when expecting a child. The strong links between relationship functioning and a wide range of adult and child outcomes have led to a growing recognition among researchers and policymakers that a happy partner relationship--which most people desire in their lives--has important public health consequences.

## Competing interests

The authors declare that they have no competing interests.

## Authors' contributions

GMBR performed the statistical analyses and drafted the manuscript. All authors contributed to the study's design, preparation of the data, interpretation of results and helped to draft or critically revise the manuscript. All authors read and approved the final manuscript.

## Pre-publication history

The pre-publication history for this paper can be accessed here:

http://www.biomedcentral.com/1471-2458/12/66/prepub
